# Supplementary material for: AddictedChem: A Data-Driven Integrated Platform for New Psychoactive Substance Identification
Source: Molecules. 2022 Jun 19;27(12):3931. doi: 10.3390/molecules27123931 (PMC9227411; doi:10.3390/molecules27123931)
Supplement: Supplementary file 1 [file molecules-27-03931-s001.zip › molecules-1625338-supplementary.pdf]

molecules

MDPI

Supplementary Materials

AddictedChem: A Data-Driven Integrated Platform for New Psychoactive Substance Identification

Mengying Han [1,†], Sheng Liu [1,†], Dachuan Zhang [1], Rui Zhang [1], Dongliang Liu [1], Huadong Xing [1], Dandan Sun [1], Linlin Gong [1], Pengli Cai [1,2], Weizhong Tu [3], Junni Chen [3] and Qian-Nan Hu [1,*]

[1] CAS Key Laboratory of Computational Biology, Shanghai Institute of Nutrition and Health, University of Chinese Academy of Sciences, Chinese Academy of Sciences, Shanghai 200031, China; hanmengying2018@sibs.ac.cn (M.H.); liusheng2019@sibs.ac.cn (S.L.); zhangdachuan2017@sibs.ac.cn (D.Z.); zhangrui2019@sibs.ac.cn (R.Z.); liudongliang2018@sibs.ac.cn (D.L.); xinghuadong2019@sibs.ac.cn (H.X.); sundandan2017@sibs.ac.cn (D.S.); gonglinlin2018@sibs.ac.cn (L.G.); caipengli@picb.ac.cn (P.C.)
[2] Tianjin Institute of Industrial Biotechnology, Chinese Academy of Sciences, Tianjin 300308, China
[3] Wuhan LifeSynther Science and Technology Co. Limited, Wuhan 430000, China; tuweizhong@163.com (W.T.); service@esynbio.com (J.C.)
* Correspondence: qnhu@sibs.ac.cn; Tel.: +86-021-54920615
† These authors contributed equally to this work.

Contents

- **Supplementary Table S1.** Targets of Schedule I drugs
- **Supplementary Table S2.** Targets of Schedule II drugs
- **Supplementary Table S3.** Targets of Schedule III drugs
- **Supplementary Table S4.** Targets of Schedule IV drugs
- **Supplementary Table S5.** Targets of Schedule V drugs
- **Supplementary Table S6.** Summary of all datasets
- **Supplementary Figure S1.** Top five scaffolds of controlled substances among different-schedule drugs
- **Supplementary Figure S2.** Gene Ontology (GO) functional annotation of interaction genes of controlled substances
- **Supplementary Figure S3.** Application of addictive compound prediction in AddictedChem

Table S1. Targets of Schedule I drugs.

UniProt (SwissProt)-recommended name of target chain	Number of targeting compounds
Sodium-dependent serotonin transporter	20
Delta-type opioid receptor	16
5-Hydroxytryptamine receptor 2B	16
5-Hydroxytryptamine receptor 2C	15
Sodium-dependent dopamine transporter	15
Cannabinoid receptor 2	13
Mu-type opioid receptor	13
Sodium-dependent noradrenaline transporter	11
Kappa-type opioid receptor	10
D(2) dopamine receptor	8
Cannabinoid receptor 1	7
5-Hydroxytryptamine receptor 6	6
Histamine H1 receptor	5
Trace amine-associated receptor 1	5
5-Hydroxytryptamine receptor 7	5
5-Hydroxytryptamine receptor 1E	4
Sigma non-opioid intracellular receptor 1	4
Alpha-1A adrenergic receptor	3
Neuronal acetylcholine receptor subunit alpha-4	3
5-Hydroxytryptamine receptor 1D	3
Histamine H2 receptor	3
5-Hydroxytryptamine receptor 3A	3
5-Hydroxytryptamine receptor 1B	3
Caspase-3	2
Muscarinic acetylcholine receptor M5	2
Neuronal acetylcholine receptor subunit alpha-2	2
Neuronal acetylcholine receptor subunit alpha-3	2
Potassium voltage-gated channel subfamily H member 2	2
Muscarinic acetylcholine receptor M3	2
Histamine H3 receptor	1
Beta-2 adrenergic receptor	1
Prostaglandin E2 receptor EP4 subtype	1
Lysosomal Pro-X carboxypeptidase	1
Proton-coupled amino acid transporter 1	1
Neuronal acetylcholine receptor subunit alpha-7	1
Beta-1 adrenergic receptor	1

Table S2. Targets of Schedule II drugs.

UniProt (SwissProt)-recommended name of target chain	Number of targeting compounds
Mu-type opioid receptor	93
Delta-type opioid receptor	83
Kappa-type opioid receptor	59
Sodium-dependent dopamine transporter	27
Sodium-dependent noradrenaline transporter	18
Sodium-dependent serotonin transporter	18
Potassium voltage-gated channel subfamily H member 2	18
Sigma non-opioid intracellular receptor 1	12
Trace amine-associated receptor 1	6
D(2) dopamine receptor	4
Synaptic vesicular amine transporter	4
Beta-2 adrenergic receptor	2
Cannabinoid receptor 1	2
Cannabinoid receptor 2	2
Lysosomal Pro-X carboxypeptidase	2
Solute carrier family 22 member 1	2
Glutamate receptor ionotropic, NMDA 1	1
Gamma-aminobutyric acid receptor subunit beta-3	1
Perilipin-5	1
Histamine H1 receptor	1
Aurora kinase A	1
Aurora kinase B	1
Perilipin-1	1
Cytochrome P450 3A4	1
Potassium voltage-gated channel subfamily A member 3	1
Hepatocyte nuclear factor 4-alpha	1
Gamma-aminobutyric acid receptor subunit alpha-1	1

Table S3. Targets of Schedule III drugs.

UniProt (SwissProt)-recommended name of target chain	Number of targeting compounds
Androgen receptor	73
Cannabinoid receptor 2	21
Cannabinoid receptor 1	18
Sex hormone-binding globulin	14
Mu-type opioid receptor	13
Kappa-type opioid receptor	13
NAD-dependent protein deacetylase sirtuin-1	11
NAD-dependent protein deacetylase sirtuin-2	9
Sigma non-opioid intracellular receptor 1	9
Delta-type opioid receptor	8
Aromatase	7
Testosterone 17-beta-dehydrogenase 3	5
Glutamate receptor 1	5
Multidrug resistance-associated protein 4	4
Cholinesterase	4
Canalicular multispecific organic anion transporter 2	4
Histamine H1 receptor	3
G-protein coupled bile acid receptor 1	3
Mineralocorticoid receptor	2
Voltage-dependent L-type calcium channel subunit alpha-1C	2
Nociceptin receptor	2
Glutamate receptor ionotropic, NMDA 1	2
D(2) dopamine receptor	1
Solute carrier family 22 member 6	1
3-Beta-hydroxysteroid-delta(8),delta(7)-isomerase	1
G-protein coupled receptor 55	1
3-Oxo-5-alpha-steroid 4-dehydrogenase 2	1
Cytochrome P450 3A4	1
Histamine H4 receptor	1
Cytochrome P450 1B1	1
N-arachidonyl glycine receptor	1
Solute carrier family 22 member 1	1
Multidrug and toxin extrusion protein 1	1

Table S4. Targets of Schedule IV drugs.

UniProt (SwissProt)-recommended name of target chain	Number of targeting compounds
Gamma-aminobutyric acid receptor subunit alpha-1	53
Gamma-aminobutyric acid receptor subunit alpha-3	34
Gamma-aminobutyric acid receptor subunit alpha-5	28
Sodium-dependent dopamine transporter	28
Mu-type opioid receptor	27
5-Hydroxytryptamine receptor 2B	21
Sodium-dependent noradrenaline transporter	20
5-Hydroxytryptamine receptor 2C	20
Sodium-dependent serotonin transporter	15
Gamma-aminobutyric acid receptor subunit alpha-6	13
Potassium voltage-gated channel subfamily H member 2	12
Multidrug resistance-associated protein 4	12
Canalicular multispecific organic anion transporter 2	12
Translocator protein	10
Orexin receptor type 2	9
Orexin receptor type 1	8
Kappa-type opioid receptor	7
Delta-type opioid receptor	6
Prostaglandin G/H synthase 2	5
Sigma non-opioid intracellular receptor 1	4
Histamine H1 receptor	4
Cholecystokinin receptor type A	4
Platelet-activating factor receptor	3
Solute carrier family 22 member 1	3
Cytochrome P450 3A4	3
Cytochrome P450 2C19	3
Carbonic anhydrase 2	3
Carbonic anhydrase 1	3
Alpha-1A adrenergic receptor	2
Albumin	2
Aldo-keto reductase family 1 member C2	2
Muscarinic acetylcholine receptor M3	2
D(2) dopamine receptor	2
Gastrin/cholecystokinin type B receptor	2
Aldo-keto reductase family 1 member C1	2
5-Hydroxytryptamine receptor 6	2
Muscarinic acetylcholine receptor M5	2
Carbonic anhydrase 9	2
Aldo-keto reductase family 1 member C3	2
Histamine H4 receptor	1
Voltage-dependent L-type calcium channel subunit alpha-1C	1
Adenosine receptor A3	1
Aldehyde oxidase	1
Carbonic anhydrase 5B, mitochondrial	1
Carbonic anhydrase 4	1
Carbonic anhydrase 3	1
Trace amine-associated receptor 1	1
Carbonic anhydrase 14	1
Proto-oncogene tyrosine-protein kinase receptor Ret	1
Carbonic anhydrase 12	1
Histamine H3 receptor	1
Cytochrome P450 1A1	1
Arachidonate 5-lipoxygenase-activating protein	1
Gamma-aminobutyric acid receptor subunit alpha-4	1
Myoglobin	1

Carbonic anhydrase 7	1
Carbonic anhydrase 5A, mitochondrial	1

Table S5. Targets of Schedule IV drugs.

**UniProt (SwissProt)-recommended name of target chain**	**Number of targeting compounds**
Sodium-dependent dopamine transporter	4
Potassium voltage-gated channel subfamily KQT member 2	4
Sodium-dependent serotonin transporter	2
Canalicular multispecific organic anion transporter 2	2
Sodium-dependent noradrenaline transporter	2
Multidrug resistance-associated protein 4	2
Potassium voltage-gated channel subfamily E member 1	1
Carbonic anhydrase 5A, mitochondrial	1
Carbonic anhydrase 1	1
Carbonic anhydrase 2	1
Carbonic anhydrase 9	1
Potassium voltage-gated channel subfamily H member 2	1
Carbonic anhydrase 12	1
Carbonic anhydrase 4	1
Carbonic anhydrase 5B, mitochondrial	1
Carbonic anhydrase 14	1
Carbonic anhydrase 3	1
Voltage-dependent L-type calcium channel subunit alpha-1C	1
Carbonic anhydrase 7	1

Table S6. Summary of all datasets.

Dataset	Source	Last accessed	Usage	Processed dataset size
DEA controlled substances	https://pubchem.ncbi.nlm.nih.gov/source/Drug%20Enforcement%20Administration%20(DEA)	13-Oct-20	Positive samples for modelling, data analysis and database construction	622
ADMET data	http://lmmd.ecust.edu.cn/admetsar2/	14-Dec-20	Database construction	821
Targets data	https://www.bindingdb.org/	30-Apr-22	Database construction	3,352
CTD pathways data	http://ctdbase.org/downloads/;jsessionid=3171C997C2B363A6F470E664B6D31FEC,	25-Jan-21	Database construction	1,102
SMPDB pathways data	http://www.smpdb.ca/downloads	16-Apr-22	Database construction	53
CTD GO data	http://ctdbase.org/downloads/;jsessionid=3171C997C2B363A6F470E664B6D31FEC,	25-Jan-21	Database construction	3,608
Cayman Chemical Company abused drugs	https://www.caymanchem.com/	14-Apr-21	Positive samples for modelling	1,880
GRAS compounds	https://www.fda.gov/food/food-ingredients-packaging/generally-recognized-safe-gras	31-Dec-20	Negative samples for modelling	173
DrugBank compounds	https://go.drugbank.com/releases/latest	12-Oct-20	Negative samples for modelling	2,329
NFLIS-Drug substances	https://www.nflis.deadiversion.usdoj.gov/nflisdata/docs/NFLISSubstanceListFormulaSorted.pdf	26-Mar-21	Positive samples for the external validation set	184
KEGG drugs	https://www.genome.jp/kegg/drug/br08319.html	2-Apr-21	Negative samples for the external validation set	148

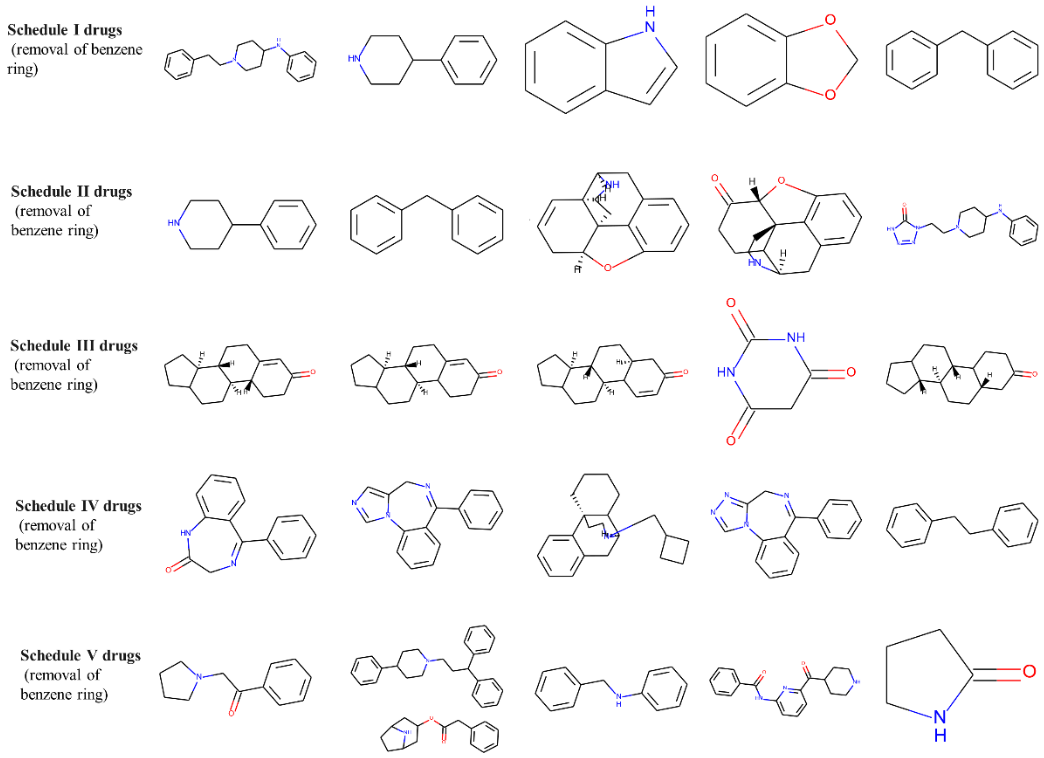

Figure S1. Top five scaffolds of controlled substances among different-schedule drugs.

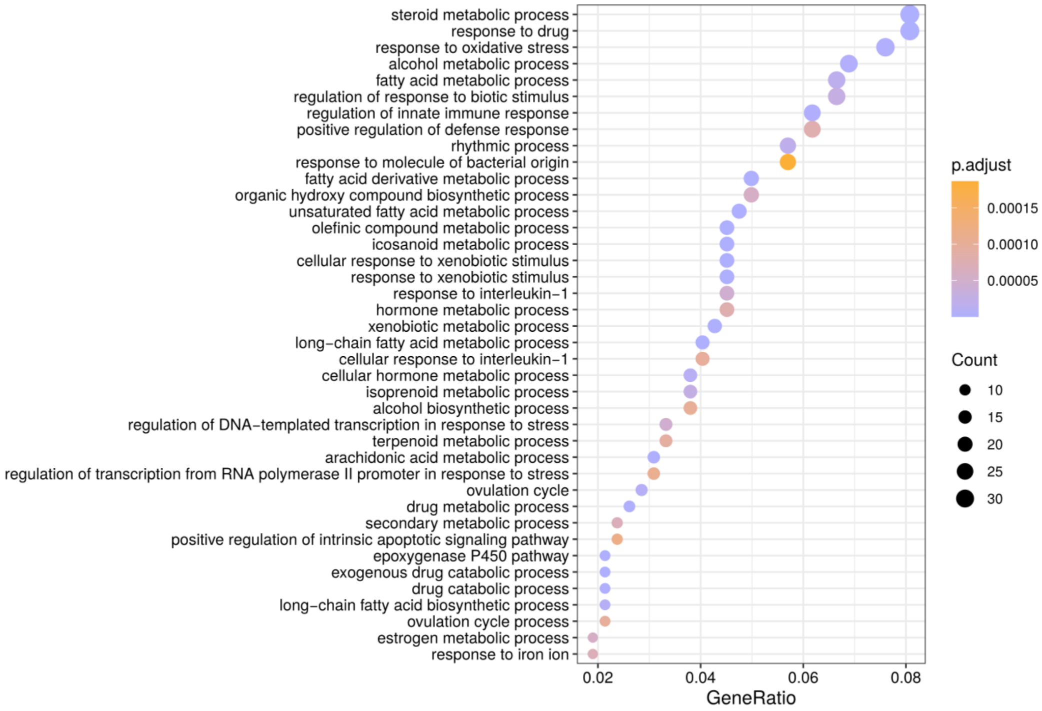

Figure S2. Gene Ontology (GO) functional annotation of interaction genes of controlled substances. GeneRatio, the ratio of the number of genes related to the GO annotation to the total number of genes. Count, the number of genes related to the GO annotation. The Figure was generated using the clusterProfiler package.

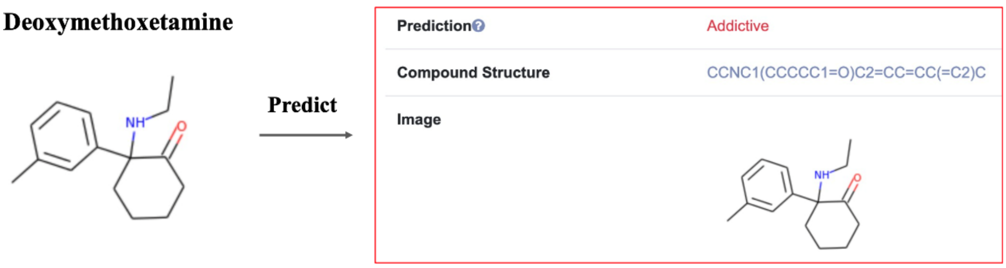

Figure S3. Application of addictive compound prediction in AddictedChem. Left: chemical structure of deoxymethoxetamine (PubChem CID: 157010705). Right: summary prediction result for deoxymethoxetamine by online platform AddictedChem. Deoxymethoxetamine is correctly predicted as NPS.
